# Effect of Asphalt Mixture Surface Preparation Methodology on Determining Luminance Level in Laboratory Conditions: Case Study in Poland

**DOI:** 10.3390/ma19071277

**Published:** 2026-03-24

**Authors:** Dominik Grzyb, Marta Wasilewska, Władysław Gardziejczyk

**Affiliations:** Faculty of Civil Engineering and Environmental Sciences, Bialystok University of Technology, Wiejska 45E Street, 15-351 Bialystok, Poland; marta.wasilewska@pb.edu.pl

**Keywords:** roads surface, luminance, glassblasting, skid resistance, macrotexture, the luminance coefficient in the diffused light

## Abstract

**Highlights:**

- The road aggregates differ significantly in terms of luminance.
- Within the same mineral–asphalt mixture, the type and grain size of the aggregate does not significantly affect the level of surface luminance.
- The glassblasting method does not fully correspond to the actual road surface.

**Abstract:**

This paper verifies a method for determining the luminance of a pavement surface made of SMA mixtures at the design stage under laboratory conditions. Tests were conducted on surfaces made of six types of SMA mixtures with varying grain sizes (between 8 and 11 mm) and coarse aggregate types like trachybasalt with a luminance coefficient in diffused light of Q_d_—53 mcd/m^2^/lx, gabbro with Q_d_—83 mcd/m^2^/lx, and granite with Q_d_—115 mcd/m^2^/lx. The effect of the glassblasting process on the changes in the luminance coefficient in diffused light (Q_d_) was analyzed while simultaneously monitoring parameters describing skid resistance and macrotexture. Additionally, it was decided that tests would be performed on two sets of specimens differing in their conditioning temperatures. It was found that conditioning at −15 °C significantly improved the binder film removal process from asphalt mixture surfaces compared to those conditioned at 22 °C. Differences were recorded between individual specimens conditioned at −15 °C at the end of the glassblasting. The lowest Q_d_ values were found for specimens with the darkest trachybasalt aggregate (SMA 8—53.0; SMA 11—51.7 mcd/m^2^/lx) and the highest for specimens with the lightest granite aggregate (SMA 8—63.9; SMA 11—59.8 mcd/m^2^/lx). However, considering the differences in Q_d_ between individual coarse aggregates, the differences between specimens with these aggregates are insignificant. Glassblasting is a cheap and quick procedure for removing a binder from the surface of specimens, preparing them for luminance determination in the laboratory. It should be noted that glassblasted surfaces should not be used to determine the skid resistance and macrotexture changes at the design stage of an asphalt mixture.

## 1. Introduction

Effective road design and management require taking action to optimize and systematize solutions for individual road infrastructure elements that will guarantee driving comfort, traffic safety, and minimize negative impacts on the environment.

The upper layer of the road pavement is responsible for driving comfort and user safety. Skid resistance plays a key role in road safety. It is defined as characterization of the friction of a road surface when measured in accordance with a standardized method [1]. Numerous studies have demonstrated a relationship between accident rates and the level of anti-skid properties of road surfaces. The risk of a skid-related accident increases by approximately 30–60% on wet and damp surfaces, depending on the road geometry [2]. The friction force depends on the surface condition, particularly on texture irregularities ranging from microtexture below 0.5 mm and macrotexture from 0.5 to 50 mm. Texture changes over the life of the road pavement due to traffic and weather conditions. These changes are related to the technology of the upper layer. The appropriate selection of binder, aggregate, asphalt mix type, and texturing technique allows for the design of a layer that meets the required level of skid resistance throughout the road’s service life. Therefore, in most countries, the criteria for selecting top layer technologies during laboratory design refer to both durability and skid resistance.

It should be noted that the choice of technology for constructing the upper surface layer is one of the factors that influences traffic safety in particularly hazardous areas. Lighting is used to ensure proper observation of the road and its surroundings by road users during periods of limited visibility, especially in urban areas. This requires uniform road illumination, proper visual guidance for drivers, and reduced glare. The luminance of individual elements, i.e., the intensity of the light impression perceived by the human eye when viewing an illuminated or luminous surface, plays a significant role [3]. The visual conditions determined by road surface luminance are important not only for drivers, but also for other road users. A crucial aspect in the context of luminance and driver perception is the visibility of a pedestrian at a crosswalk. This depends largely on the luminance of the background, which, in this case, is the road surface [4]. A previous study showed that the level of luminance contrast between a pedestrian’s silhouette and the road surface is a key factor determining their visibility to drivers [5]. At the same time, the authors emphasize that simply increasing illuminance does not guarantee improved visibility if it is accompanied by an increase in background luminance, leading to a reduction in contrast. This confirms that, in addition to increasing illuminance, attention should be paid to the photometric properties of the road surface. Because the upper surface layers are constructed using different technologies and materials, they exhibit varying luminance properties. Therefore, the parameters describing road surface luminance were considered in lighting design as early as the 1980s.

In 1984, the International Commission on Illumination (CIE) introduced the R-classification system in which road surfaces were assigned to one of four categories, from R1 to R4. For each category, a table was developed containing the values of the reduced luminance coefficient, depending on the angle of light incidence and the angle of observation [6]. It should be noted that in situ measurements were not performed. Specimens were cut from the road’ surface and were tested using goniophotometers in the laboratory [7]. The classification was developed based on measurement data obtained in the 1970s; progress in the development of materials and technologies used for road surfaces is significant.

Currently, several types of asphalt mixtures (dense-graded, gap-graded, open-graded) are used that influence the surface texture. Consequently, they are characterized by varying luminance. Research conducted in the United Kingdom has shown that the type of asphalt mixture influences the road luminance distribution [6]. However, this analysis did not take into account the texture. In Poland, mathematical models have been developed to predict the luminance level of both aggregates and wearing courses made of asphalt mixtures [8,9]. Similar studies were conducted in Germany, Finland, and Belgium. Their objective was to determine the influence of the luminance coefficient of coarse aggregates intended for wearing courses on their brightening [10]. However, these studies did not take into account the physical properties of materials, especially aggregates, which significantly influence characteristics such as skid resistance, macrotexture, and microtexture. Eren et al. examined both the reflective properties surface using a series of photometric indicators, such as average luminance coefficient, specularity factor, and reflection factor, and measures describing macrotexture based on the MTD (Mean Texture Depth) and skid resistance based on the PTV (Pendulum Tester Value). It was indicated that, when selecting materials and treatments to improve surface brightness, other surface characteristics should be verified. Because there are some surface treatments, e.g., those containing only white polymers, which cause a reduction in the macrotexture depth and a decrease in the microtexture parameters [11]. However, these studies focused on surface treatment and the assessment of surface properties in laboratory conditions, but without simulating the surface wear that occurs under traffic and weather conditions. However, a study conducted in Lithuania demonstrated a relationship between changes in the luminance and the type of wearing course and its service life [12].

In many countries, it is recommended to use light-colored aggregates due to their brightening effect on the surface [13]. Such solutions have been proven to improve safety. Erkan et al. conducted research on the minimum required road surface luminance level in relation to driving speed. They found that the higher the road surface luminance level, the better the perception of brightness, a greater sense of driver safety, and an improved reaction time. [14,15,16]. Aggregates are produced from various types of rock. The properties of the rocks influence the geometric and physical properties of fine and coarse aggregates. Unfortunately, the raw material base for aggregate production is limited. This contributes to the search for alternative solutions. Scientists, understanding the impact of luminance on lighting costs, are striving to find unconventional materials to reduce lighting energy demand while maintaining visibility standards [17,18,19]. The topic is especially important in tunnel surfaces, where artificial lighting plays a significant role. Gu et al. analyzed binder materials including polyurethane, transparent and light-colored asphalt, and two aluminate-based long afterglow materials, including long-afterglow luminescent gravel (LALG) and luminescent powder (LP). The self-luminous pavement material exhibited comparable mechanical performance to the conventional asphalt mixture, but improved the luminance coefficient, which would save 66.7% light energy in a municipal road [20]. The use of polymer-modified coatings and epoxy-based binders allows for the creation of reflective surfaces. Studies have shown their wear resistance compared to conventional dark asphalt sealants [21,22].

In Poland, in 2014, a mandatory requirement was introduced to monitor the luminance of coarse aggregates in asphalt mixtures intended for wearing courses and their surfaces in laboratory conditions. The luminance coefficient in diffused light Q_d_ was adopted as the luminance measure. Wearing courses on expressways should have a Q_d_ ≥ 70 mcd/m^2^/lx, and in tunnels, Q_d_ ≥ 90 mcd/m^2^/lx. This parameter was implemented in the 1990s as an alternative to in situ luminance assessment of road markings. It refers to the luminance of a given object’s surface under conditions similar to diffuse lighting. Introducing this parameter into the monitoring system allows for the control of the properties of road markings from the driver’s perspective in weak daylight conditions (cloudy day, twilight) [23]. A reflectometer or a retroreflectometer measures the Q_d_ coefficient under established conditions, i.e., an observation angle of 2.29°, which corresponds to the observation distance from the driver’s eye level at a distance of 30 m [24]. In 2000, the EN 1436 standard was issued, which, together with subsequent revisions, specifies the measurement methods [25]. It should be noted that European countries still use the R-classification system and not the Q_d_ parameter. Additionally, there are few publications that present the findings of the surface preparation of specimens made from asphalt mixture for laboratory luminance assessment. It should be noted that there is no method for determining luminance at the stage of designing the composition of asphalt mixtures, which would be described in a normative document. Polish Technical Guidelines—WT-2 apply only to national roads. It is not a legal act or standardization document that provides rules or guidelines for common use. The lack of a normative document hinders the comparisons of different solutions and the exchange of experiences between countries.

Stationary devices for measuring Q_d_ are easy to use and allow for both laboratory and in situ measurements. The method of surface preparation for determining luminance in the laboratory is crucial. This is due to the presence of a binder film on the aggregate of asphalt mixtures compacted in laboratory conditions. This film is worn away by vehicle traffic, fine dirt, and water in real-world road conditions. The abrasion and polishing phenomena intensify in the tracks of vehicle wheels. This leads to differences in macrotexture and microtexture across the lane cross-section and, therefore, in the skid resistance and luminance distribution on the surface. Numerous publications have highlighted the role of macrotexture in light reflection: it helps to evenly disperse light, reduce glare, and, consequently, improve visual comfort, especially in tunnels, where uniformity of lighting is important [26]. Therefore, it is crucial to correctly simulate abrasion and polishing phenomena to obtain a comparable level of texture to that observed in actual conditions. Comparison was made between the light-reflecting quantities measured on specimens after different treatment methods in laboratory conditions and those measured on the road surface in situ. Results comparable to in situ conditions were obtained by sawing and lightly sandblasting samples made in gyratory press. [27]. In practice, devices for simulating traffic conditions are primarily used to assess the variability of skid resistance and macrotexture. The Friction After Polishing (FAP) machine, the Model Mobile Load Simulator (MMLS3), and other cyclic polishing machines are well-described in the literature [28,29]. However, these tests are not widely used, and the number of laboratories with these devices is limited. There is no available source data indicating that they could be used to verify luminance. In Poland, asphalt mixture specimens on which Qd measurements are performed must be subjected to glassblasting, i.e., sandblasting using glass microspheres [30]. This method can be performed with specimens [27], but it interferes with the surface texture of the tested specimen. Therefore, both macrotexture and microtexture should be monitored in laboratory tests.

The objective of this study was to determine the effect of the glassblasting process of the SMA (Stone Mastic Asphalt) mixture surface on the values of the luminance coefficient in diffused light Q_d_ while simultaneously monitoring parameters describing microtexture and macrotexture. Due to the influence of temperature on binder properties, a decision was made to determine the effect of the specimen conditioning temperature during glassblasting.

## 2. Materials and Methods

### 2.1. Materials and Tested SMA Mixtures

The tested SMA11 and SMA8 mixtures were designed according to [30,31]. SBS modified binder PMB 45/80-55 and coarse aggregates of varying luminance were used. The properties of the coarse aggregates are presented in Table 1. The following were selected:

Coarse aggregate from extrusive trachybasalt rock, marked as Qd-1, with the lowest average value of the luminance coefficient in diffused light $\bar{Q_{d}}$, equal to 53 mcd/m^2^/lx;
Coarse aggregate from excavated gabbro rock, marked as Qd-2, with an indirect average value of the luminance coefficient in diffused light $\bar{Q_{d}}$, equal to 83 mcd/m^2^/lx;
Coarse aggregate from deep granite rock, marked as Qd-3, with the highest average value of the luminance coefficient in diffused light and the highest average value of the luminance coefficient in diffused light $\bar{Q_{d}}$, equal to 115 mcd/m^2^/lx.

**Figure 1:**
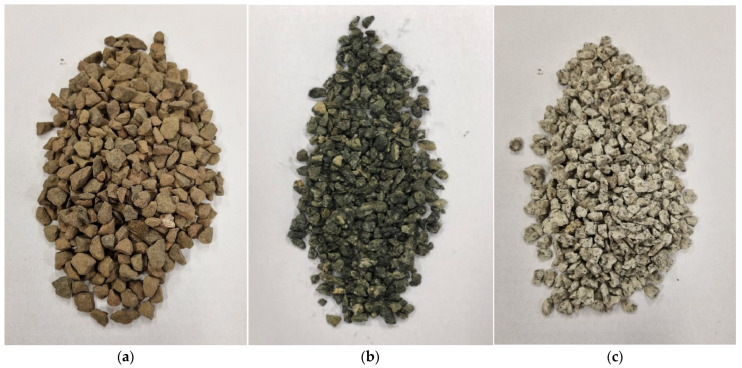
Coarse aggregate (**a**) trachybasalt (Qd-1); (**b**) gabbro (Qd-2); (**c**) granite (Qd-3).

**Figure 2:**
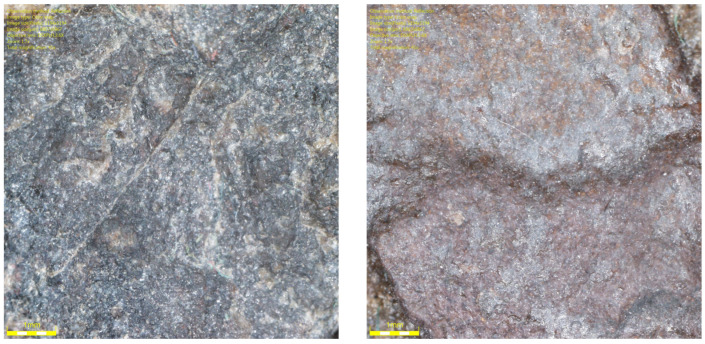
Image of trachybasalt aggregate grains (Qd-1) $\bar{Q_{d}}$—53 mcd/m^2^/lx, x41.

**Figure 3:**
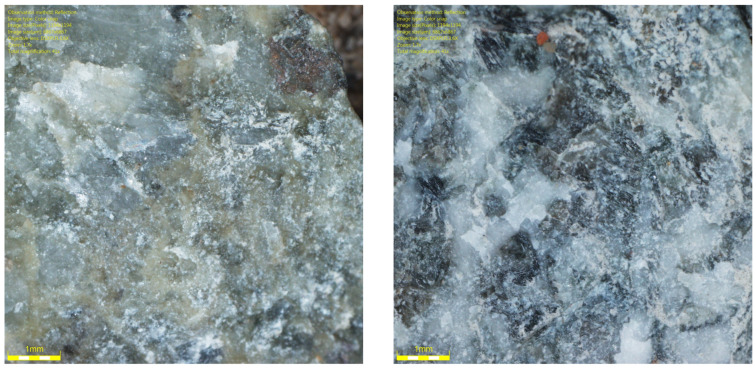
Image of gabbro aggregate grains (Qd-1) $\bar{Q_{d}}$—83 mcd/m^2^/lx, x41.

**Figure 4:**
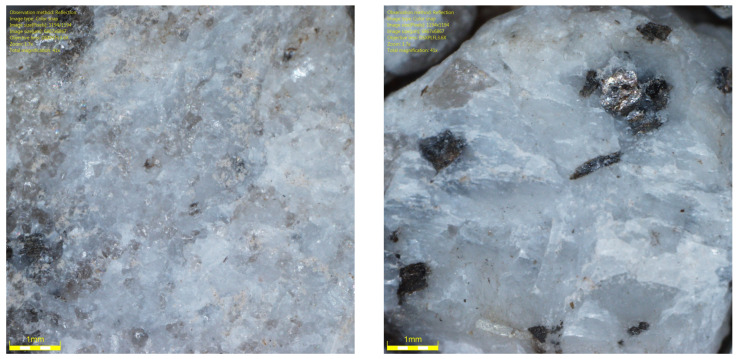
Image of granite aggregate grains (Qd-3) $\bar{Q_{d}}$—115 mcd/m^2^/lx, x41.

Figure 1 shows the coarse aggregates, and Figure 2, Figure 3 and Figure 4 show their sample surfaces under an optical microscope.

**Figure 5:**
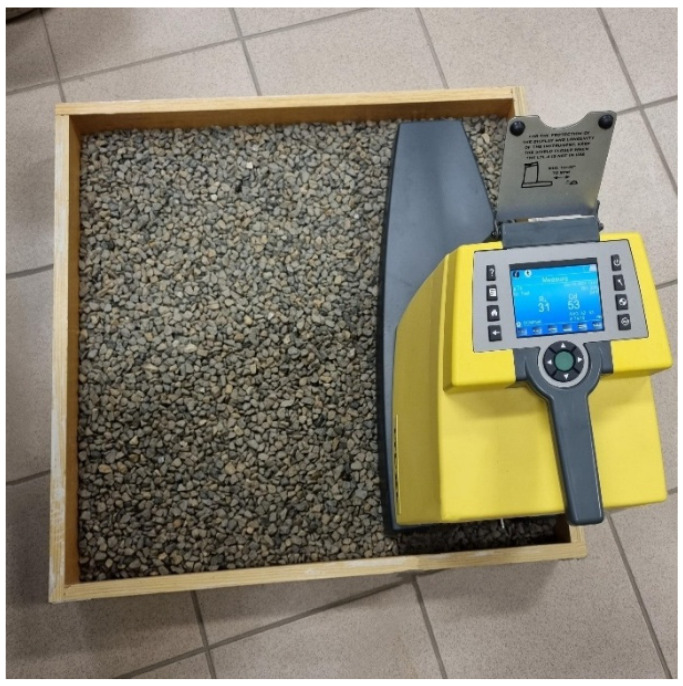
Representative measurement of the aggregate Q_d_ coefficient.

Aggregate luminance was measured using a retroreflectometer LTL-XL (DELTA/RoadSensors). This involved filling a special mold with aggregate, compacting the sample, and taking measurements (Figure 5).

The mixtures were designed to have comparable void content (Vb ≈ 3%) and coarse aggregate content (75–80%). The grading is presented in Table 2. Asphalt mixture specimens were made in 500 × 400 × 50 mm format and compacted in accordance with EN 12697-33 [37]. Five specimens were made in the form of a slab for each mixture. Three specimens from each asphalt mixture were randomly selected for testing.

The volumetric parameters of SMA8 and 11 mixtures are presented in Table 3.

### 2.2. Experimental Procedure

**Figure 6:**
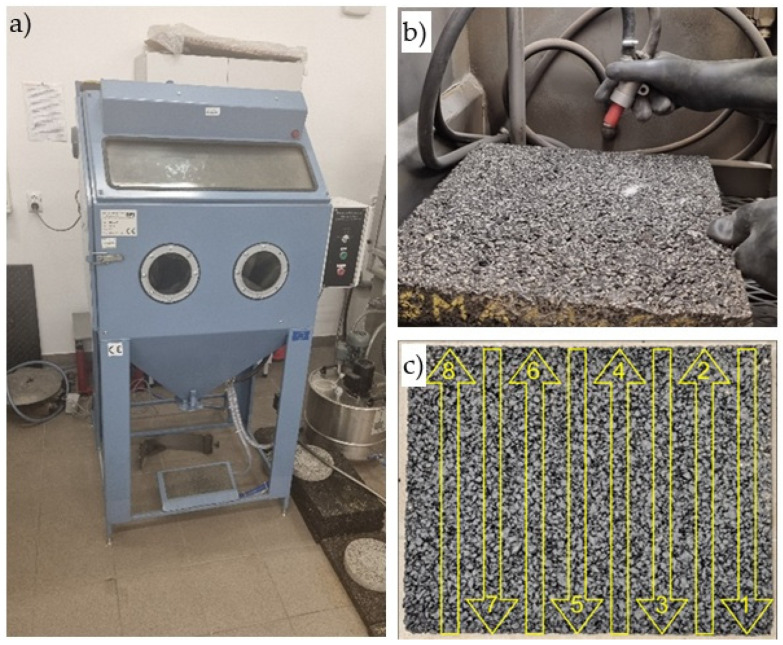
Glassblasting process: (**a**) glassblasting chamber, (**b**) abrasive feeding process using a dosing nozzle, and (**c**) nozzle operation diagram during one glassblasting cycle.

**Figure 7:**
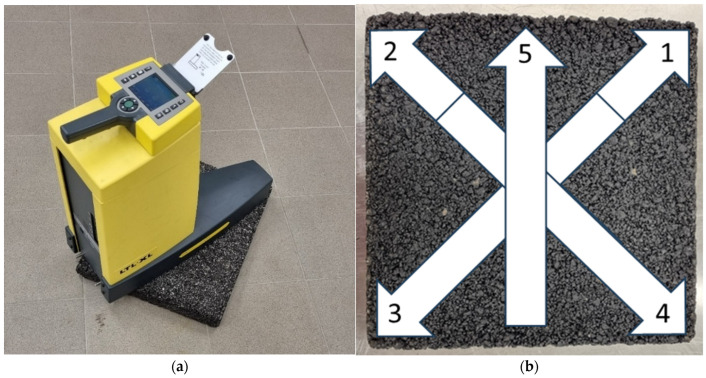
(**a**) Measurement of Qd; (**b**) retroreflectometer setting diagram on the surface of the specimen.

To verify the effect of conditioning temperature on the luminance coefficient in diffused light Q_d_, samples were conditioned at room temperature, 22 °C (marked with the symbol N—unfrozen), and frozen at −15 °C (marked with the symbol M—frozen). The conditioning process involved storing the samples at a given temperature for 24 h before each glassblasting cycle in a refrigerated cabinet. Glassblasting was performed in a chamber (Figure 6a) using glass microspheres with a granulation of 0.3–0.4 mm. These were applied to the specimen surface using a dispensing nozzle at a pressure of 8 bar (Figure 6b). The operator’s movement of the dispensing nozzle across the sample during one cycle is shown in Figure 6c. Because one nozzle movement cycle, which lasted approximately 6 min, did not completely remove the binder film, the cycles had to be repeated. After each glassblasting cycle, the specimens were washed and dried at 40 °C for 20 h. Only then were the luminance coefficient Q_d_ measured using the LTL-XL retroreflectometer (DELTA/RoadSensors). The device was positioned in the center of the specimen and five readings were taken, each time with the retroreflectometer facing a different direction (Figure 7).

**Figure 8:**
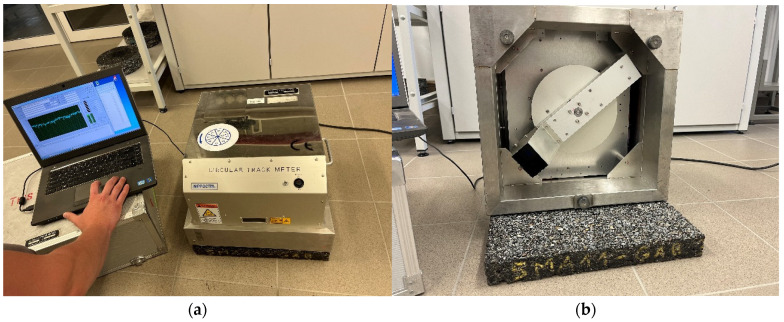
(**a**) CTM device; (**b**) bottom view.

**Figure 9:**
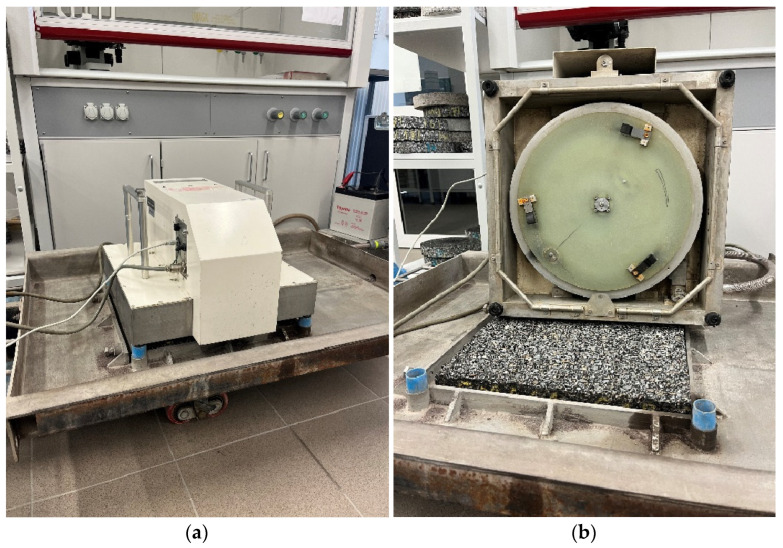
(**a**) DFT device; (**b**) bottom view.

A CTM Circular Track Meter laser profilograph (Figure 8) was used to assess the macrotexture in accordance with [41]. The device measures the MPD (Mean Texture Depth) parameter using a CCD laser displacement sensor moving around a 284 mm diameter circle, which records the surface profile. Changes in the microtexture were determined based on the DFT20 coefficient of friction using a DFT Dynamic Friction Tester (Figure 9) in accordance with [42].

In addition, after each glassblasting cycle, an image of the surface was taken using an optical microscope, which allowed for the assessment of the degree of binder film removal from the aggregate grain surface. Conditioning and glassblasting were terminated when no changes in the aggregate exposure were observed on the specimen’s surface. A total of nine glassblasting cycles were performed on each specimen. Preliminary tests conducted over 12 cycles showed that changes in values after 10 blasting sessions included visible damage to the shape of coarse aggregate grains and loss of fine aggregates. Therefore, 9 cycles were adopted.

## 3. Results and Analysis

### 3.1. Changes in the Luminance Coefficient of Diffused Light Q_d_ During the Glassblasting Process

**Figure 10:**
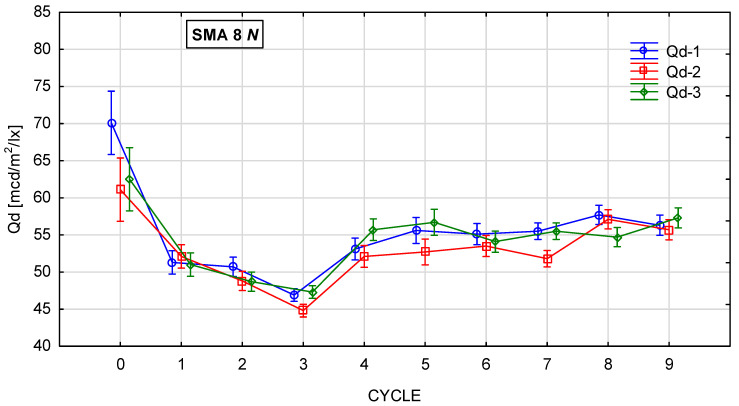
Q_d_ changes in SMA 8 N conditioned at 22 °C during glassblasting.

**Figure 11:**
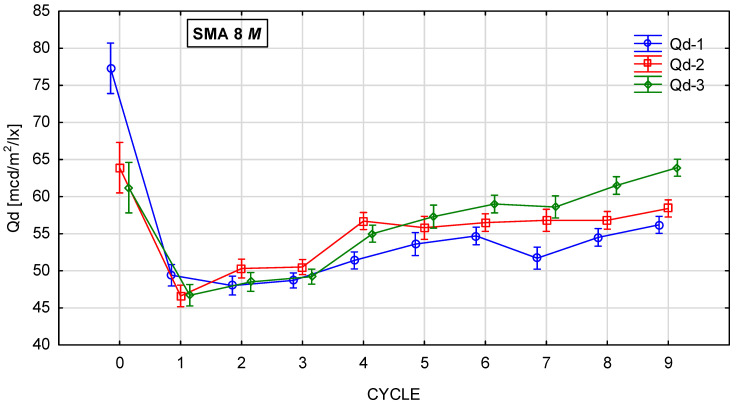
Q_d_ changes in SMA 8 M conditioned at −15 °C during glassblasting.

**Figure 12:**
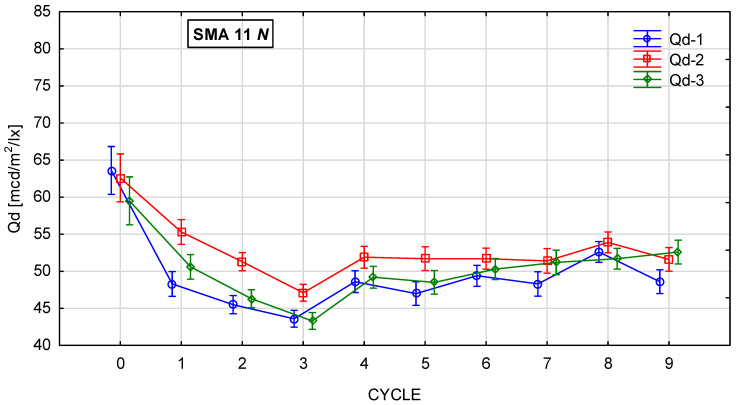
Q_d_ changes in SMA 11 N conditioned at 22 °C during glassblasting.

**Figure 13:**
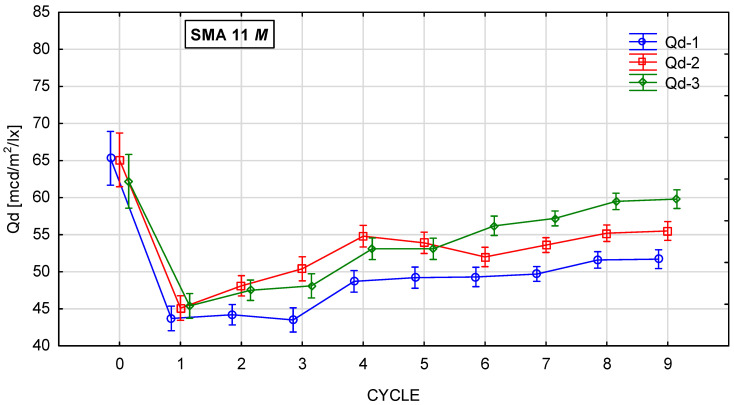
Q_d_ changes in SMA 11 M conditioned at −15 °C during glassblasting.

Figure 10, Figure 11, Figure 12 and Figure 13 show the changes in the value of the luminance coefficient in diffused light Qd in the process of glassblasting of specimens made of SMA 8 and SMA 11 mixtures, depending on their conditioning temperature. The mean value Q_d_ and standard deviation were obtained from 15 measurements obtained on three slabs independently subjected to glassblasting.

**Figure 14:**
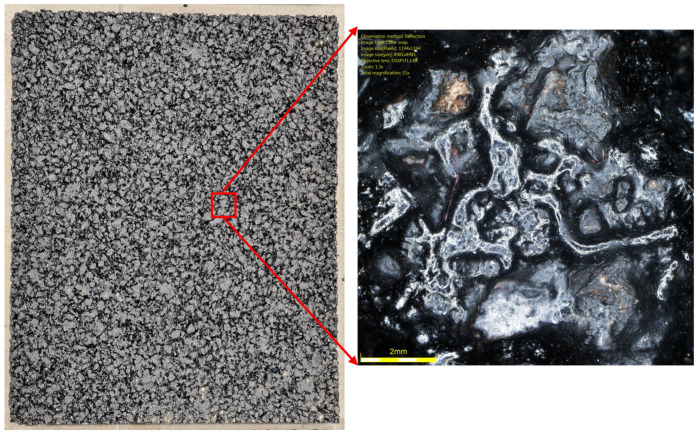
Surface of an example SMA 8 specimen before glassblasting.

The highest luminance coefficient values Q_d_ were obtained before the glassblasting process on specimens conditioned at negative and positive temperatures. This was caused by the smoothing of the asphalt mixture surfaces by the plate compactor heads. The binder film on the aggregate surface behaved like a mirror. This distorted the results, overestimating their values (Figure 14). The same effect can be observed when the surface is wet. This demonstrates the validity of performing tests only on dry surfaces.

**Figure 15:**
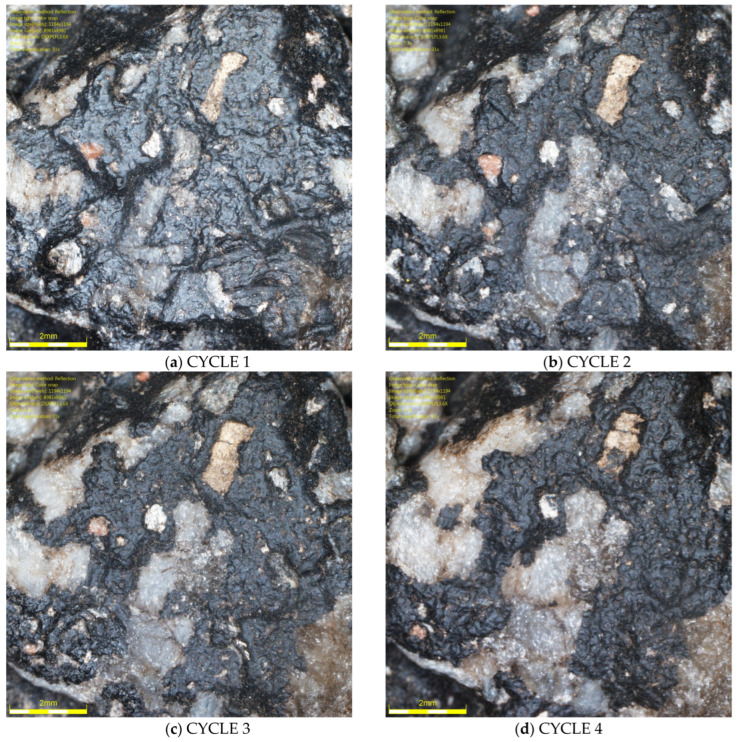
Image of granite aggregate grain (Q_d_-3) specimen of the SMA 8 N after (**a**) 1, (**b**) 2, (**c**) 3, and (**d**) 4 cycles.

It was found that there were differences in the trends in Q_d_ values during glassblasting depending on the conditioning temperature of the asphalt mixture specimens. For the surfaces of unfrozen SMA N specimens, it was observed that, in cycles 1–3 (Figure 15a–c), only the upper surface of the binder film was removed, but without exposing the aggregate grains. This resulted in a dulling of the binder surface and a decrease in the Q_d_ value. After cycle 3, the lowest Q_d_ values were obtained (43–47 mcd/m^2^/lx). As a result of subsequent glassblasting cycles, the binder film was removed from larger areas of the aggregate. This led to the gradual exposure of the grain surfaces and, consequently, an increase in the Q_d_ value (15d). No significant effect of the aggregate luminance level was observed on the luminance level of individual specimens from SMA 8 N and SMA 11 N conditioned at 22 °C. Furthermore, the differences between the mean Q_d_ values, both between individual cycles and between SMA 8 N and SMA 11 N, were insignificant. Even after the ninth glassblasting cycle, the results obtained on the surface of the SMA 8 N mixture were at the same level (56–57 mcd/m^2^/lx). In the case of SMA11 N, these differences were slightly higher. After the ninth cycle, the SMA11 N mixture with a trachybasalt aggregate (Q_d_-1) is characterized by the lowest Q_d_ value (49 mcd/m^2^/lx), while SMA 11 N with a granite aggregate (Q_d_-3) has the highest (53 mcd/m^2^/lx). Taking into account the macroscopic and microscopic evaluation of the surface, the randomness of the obtained results cannot be ruled out.

**Figure 16:**
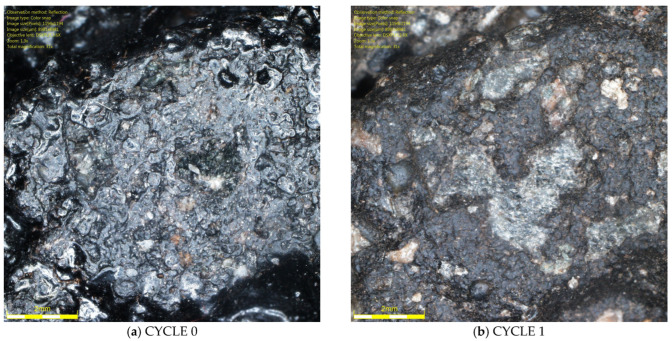

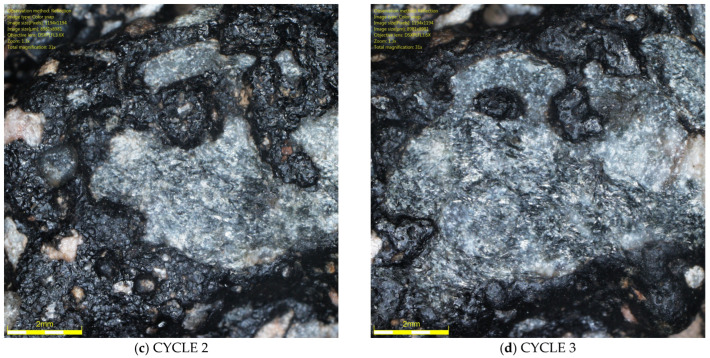
Image of gabbro aggregate grain (Q_d_-2) specimen of the SMA 8 M (**a**) before the test and after (**b**) 1, (**c**) 2, and (**d**) 3 cycles.

Conditioning SMA 8 M and 11 M specimens at a negative temperature of −15 °C significantly reduced the affinity of the aggregate and binder. Consequently, this accelerated the process of exposing aggregate grains from the binder compared to specimens conditioned at a positive temperature of 22 °C. The lowest Q_d_ values were obtained after the first cycle (44–50 mcd/m^2^/lx) (Figure 16a,b). However, after the second cycle, they gradually increased due to the exposure of the aggregate grain surface (Figure 16c,d). There is a visible relationship between the average value of the luminance coefficient in the diffuse light recorded on the surface of a given coarse aggregate and the average luminance coefficient in the diffused light of the surfaces of the tested asphalt mixtures. In the case of SMA 8 M, this occurred after the fifth cycle, and in the case of SMA 11 M, after the sixth cycle of glassblasting. At the end of the tests, the specimens based on the darkest trachybasalt aggregate (Q_d_-1) obtained the lowest values (SMA 8 M—56 mcd/m^2^/lx, SMA 11 M—52 mcd/m^2^/lx), and those based on the lightest granite aggregate (Q_d_-3) obtained the highest values (SMA 8 M—64 mcd/m^2^/lx; SMA 11 M 60 mcd/m^2^/lx). The Q_d_ of asphalt mixtures with a gabbro aggregate (Q_d_-2) is between these results (SMA8M—58 mcd/m^2^/lx; SMA 11 M—56 mcd/m^2^/lx). SMA 8 M is characterized by higher Q_d_ values than SMA 11 M. These differences average 2 units higher for mixtures with gabbro aggregate (Q_d_-2) and 4 units higher for mixtures with the trachybasalt (Q_d_-1) and granite (Q_d_-3) aggregate. This is due to differences in the mineral composition of SMA 8 M and SMA 11 M. Table 4 presents the surfaces of individual SMA mixtures after glassblasting. Both macroscopically and microscopically, it was observed that the surface of aggregate grains less than 8 mm was fully exposed. However, on the surface of grains with a diameter of approximately 11 mm, the binder still remained in microirregularities.

**Figure 17:**
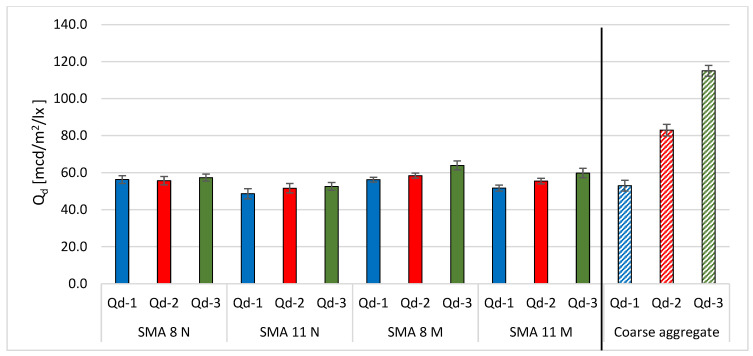
Comparison of average Q_d_ of tested surfaces after glassblasting process.

Figure 17 compares the results obtained after the final glassblasting of SMA mixture surfaces. Additionally, the results for coarse aggregates are included. Specimens conditioned at a negative temperature of −15 °C are characterized by lower average values of the luminance coefficient in diffused Q_d_ after glassblasting than specimens stored at a positive temperature of 22 °C. This is due to the fact that the binder film is removed more quickly from the surface of frozen plants. This leads to improved results. Specimens from the asphalt mixture should be conditioned at a negative temperature before glassblasting the surface to determine luminance. However, the effect of the aggregate luminance level on the luminance level of SMA 8 and 11 specimens is insignificant. The difference between SMA with the darkest trachybasalt aggregate and SMA with the lightest granite aggregate is approximately 7–8 mcd/m^2^/lx. However, the difference between the luminance coefficients determined on the surface of these aggregates is 62 mcd/m^2^/lx.

### 3.2. Changing the Surface Texture

**Figure 18:**
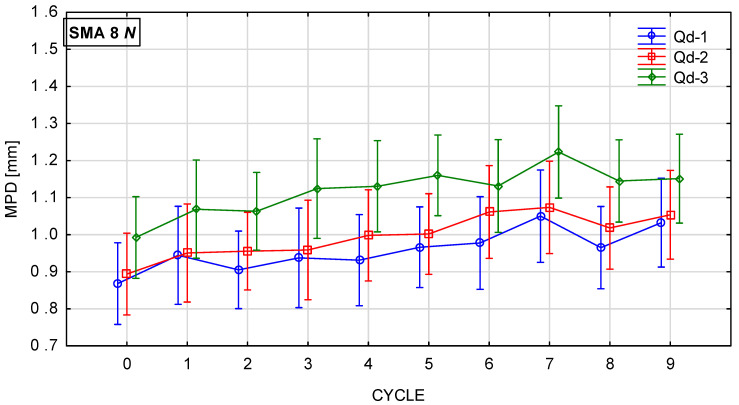
MPD changes in SMA 8 N conditioned at 22 °C during glassblasting.

**Figure 19:**
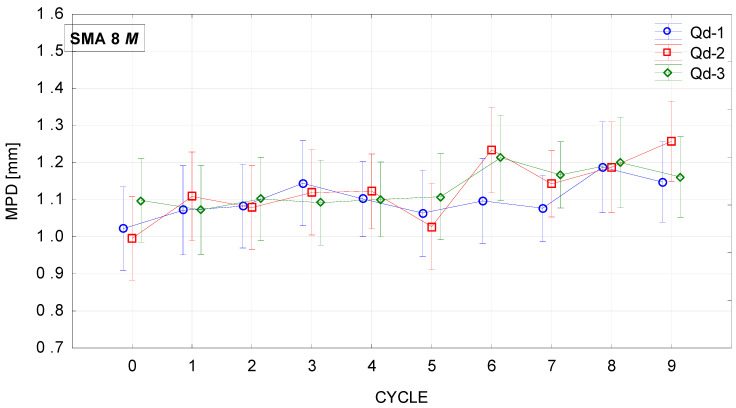
MPD changes in SMA 8 M conditioned at −15 °C during glassblasting.

**Figure 20:**
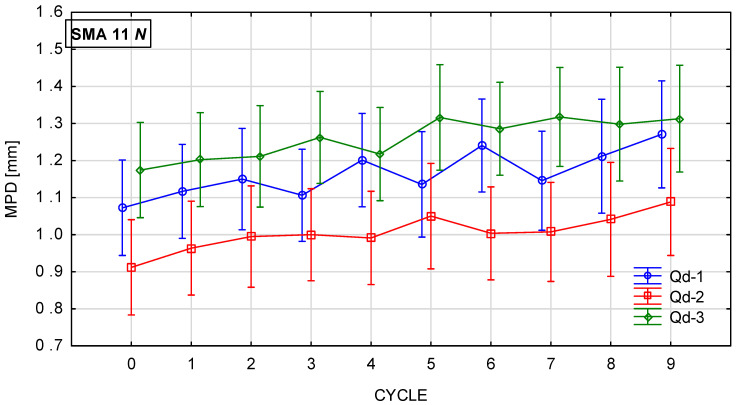
MPD changes in SMA 11 N conditioned at 22 °C during glassblasting.

**Figure 21:**
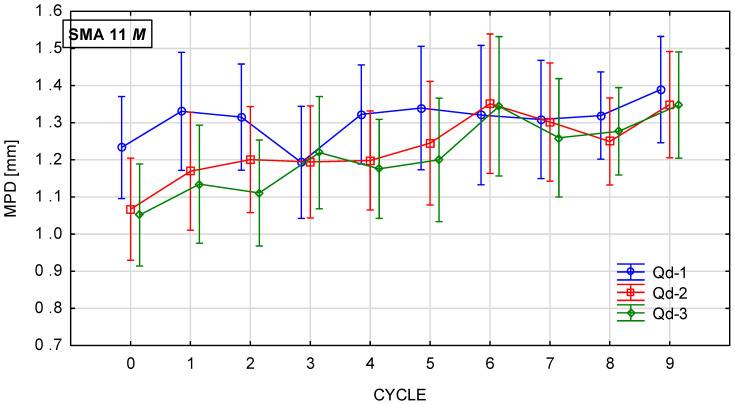
MPD changes in SMA 11 M conditioned at −15 °C during glassblasting.

Figure 18, Figure 19, Figure 20 and Figure 21 show changes in the MPD parameter value during the glassblasting process of specimens made of SMA 8 and SMA 11 mixtures depending on their conditioning temperature. The mean value and standard deviation were obtained from nine measurements obtained on three slabs subjected to separate glassblasting.

Wearing courses made of SMA 8 and SMA 11 are characterized by different levels of average depth of texture profile due to the maximum size of aggregate grains and the technical condition of the road surface [43]. If aggregate loss and cracks are not observed on the surface, the macrotexture is primarily related to the grain size of the mineral mixture [44]. Before the glassblasting process, the influence of the asphalt mixture composition on the MPD value is visible. These values are higher on SMA11 surfaces than on SMA8 surfaces. Differences were also noted between SMAs with the same maximum aggregate size but a different coarse aggregate. Despite the fact that the grain size of individual SMA8 mixtures is compared, this may indicate that the slight differences between them are related to the compaction process of the slab made from a given mixture. The same applies to specimens made of SMA11.

Regardless of the specimen conditioning method, the mean MPD values between cycles were variable, with a slightly increasing and decreasing tendency. This is related to the action of compressed air-driven glass beads to expose aggregate grains from the binder. As a result, an increase in the mean profile depth was recorded after the ninth cycle compared to the value before the glassblasting process. However, the MPD of the SMA 8 M surface with each type of aggregate is comparable after the sixth cycle. Similar trends are observed for SMA 11 M mixtures. However, mixtures with the same maximum aggregate size, differing only in the aggregate used, conditioned at 22 °C, exhibit different values of MPD. This proves the validity of conditioning specimens at negative temperatures.

**Figure 22:**
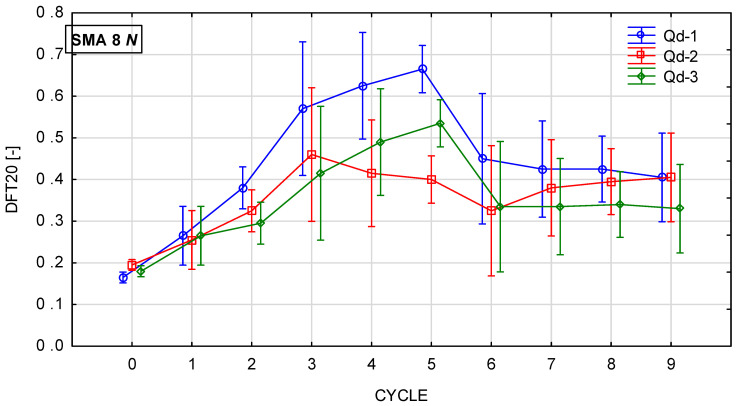
DFT20 changes in SMA 8 N conditioned at 22 °C during glassblasting.

**Figure 23:**
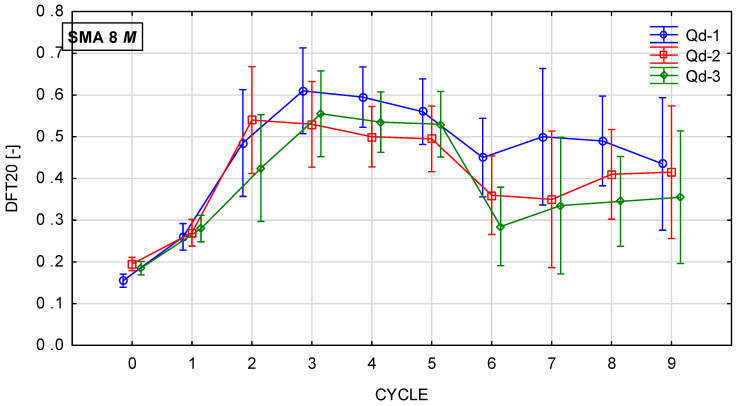
DFT20 changes in SMA 8 M conditioned at −15 °C during glassblasting.

**Figure 24:**
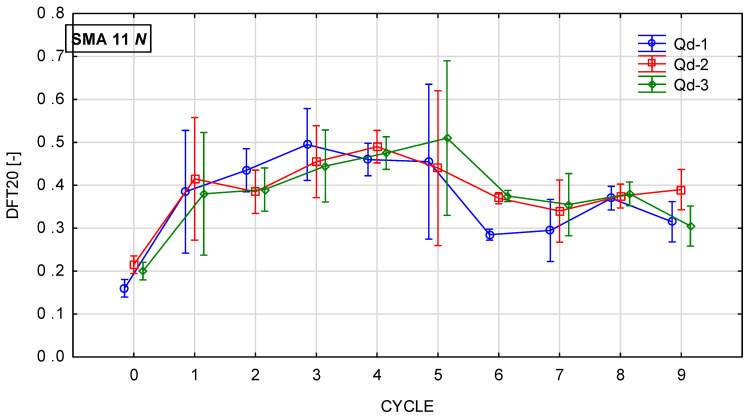
DFT20 changes in SMA 11 N conditioned at 22 °C during glassblasting.

**Figure 25:**
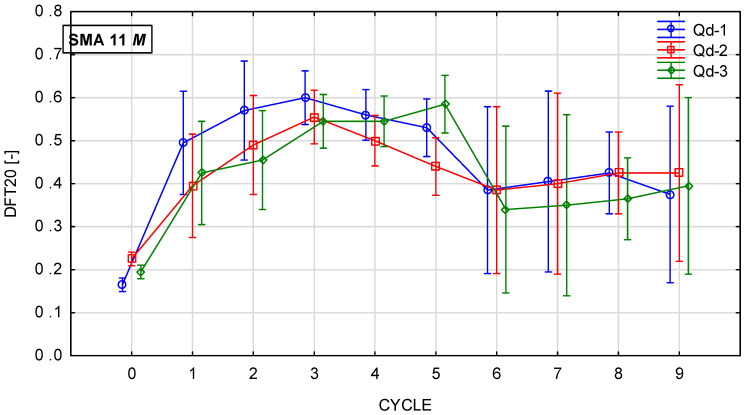
DFT20 changes in SMA 11 M conditioned at −15 °C during glassblasting.

Glassblasting affects the microtexture much more strongly. Figure 22, Figure 23, Figure 24 and Figure 25 present th changes in the DFT20 coefficient of friction during the glassblasting process for specimens made of SMA 8 and SMA 11 mixtures, depending on their conditioning temperature. The mean value and standard deviation are obtained from nine measurements obtained on three slabs subjected to separate glassblasting.

Before the glassblasting, the lowest DFT20 values (from 0.17 to 0.22) were obtained. This is a result of slipperiness occurring on a freshly laid SMA wearing course. In real road conditions, 2/4 mm aggregate is used to compact the wearing course. This protects the surface from slipperiness and accelerates the exposure of coarse aggregate grains from the binder film due to the impact of 2/4 grains being torn off by the impact of car tires.

An increase in DFT20 was observed for SMA 8 M specimens conditioned at −15 °C between the first and third cycles. Apart from mixtures with gabbro aggregate (Q_d_-2), specimens with SMA 8 N conditioned at 22 °C are characterized by an increasing tendency up to the fifth cycle. This was due to the slower rate of binder removal. After the fifth cycle, the binder film was still removed from the aggregate surface, but the action of the abrasive on the already exposed areas of the aggregate grains caused changes in its microtexture. This resulted in a reduction in the DFT20 values of specimens conditioned at both positive and negative temperatures.

Despite the range in the mean results of the SMA 8 M between cycles 6 and 8 (from 0.29 to 0.50) and after cycle 9, the DFT20 values for all mixtures differed slightly. This may be due to the characteristics of the glassblasting. Polishing, unlike in simulations of the phenomenon occurring in laboratory devices for assessing skid resistance, does not occur—only abrasion [45]. This means that, even with a large number of glassblasting cycles, the DFT20 friction coefficient values obtained on specimens with different aggregates are comparable. This masks the influence of the aggregate’s polishing resistance. Similar trends are observed for mixtures with a maximum aggregate size of 11 mm. Specimens conditioned at subzero temperatures with trachybasalt (Q_d_-1) and gabbro (Q_d_-2) aggregates exhibited the highest DFT20 values after the third cycle, while granite (Q_d_-3) aggregates exhibited the highest DFT20 values after the fifth cycle. The high standard deviations in cycles 5 and 6 are due to uneven removal of the binder film. The same trends were observed for SMA11M specimens as for SMA 8M specimens. The mean DFT20 values were similar after the ninth cycle.

**Figure 26:**
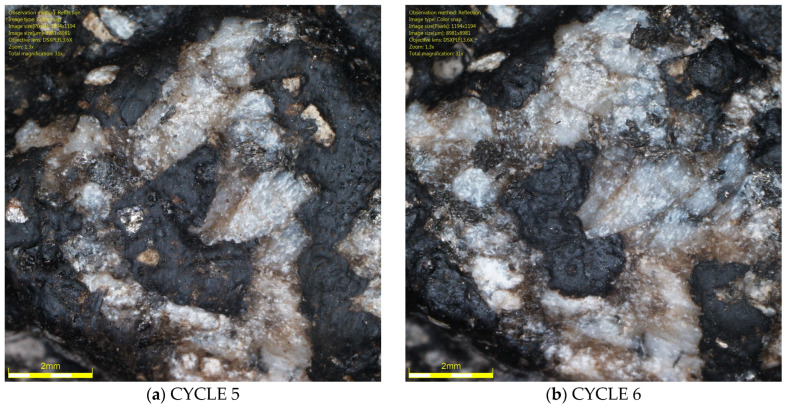
Granite aggregate grain image (Q_d_-3) on the SMA 8 M surface after (**a**) 5 I and (**b**) 6 cycles.

**Figure 27:**
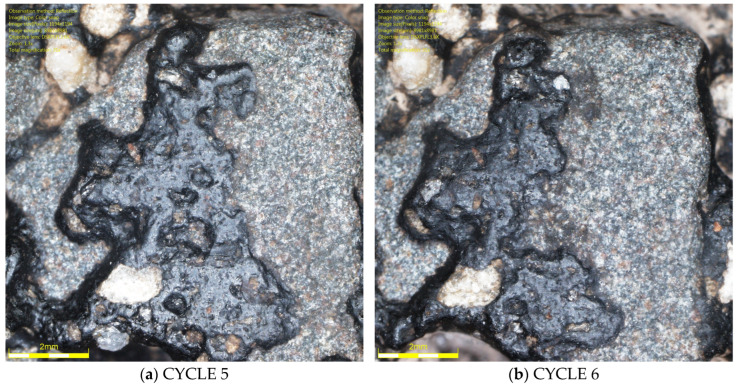
Trachybasalt aggregated grain image (Q_d_-1) on the SMA 8 M surface after (**a**) 5 I and (**b**) 6 cycles.

It should be noted that the range results for the luminance coefficient in diffused light Q_d_, the MPD parameter, and the DFT20 friction coefficient indicate a technological limitation associated with glassblasting. Despite efforts to operate the abrasive material dispensing nozzle with the highest precision, it is impossible for the operator to maintain a constant height and speed of the nozzle over the sample surface throughout the glassblasting cycle. This may result in uneven removal of the binder film from the specimens’ surfaces. Differences in the rock structure from which the aggregates were formed, and differences in the thickness of the binder film between individual grains cannot be ruled out. This is evidenced by the surfaces of some grains between cycles 5 and 6, as shown in Figure 26 and Figure 27.

In the case of the granite aggregate (Figure 26), the binder film was more difficult to remove than in the case of the trachybasalt aggregate (Figure 27). This is likely related to the open-crystalline structure of the granite. Numerous micro-irregularities and an irregular surface improve the aggregate’s affinity for the binder. Trachybasalt has a partially crystalline structure without irregularities resulting from the presence of larger minerals.

**Figure 28:**
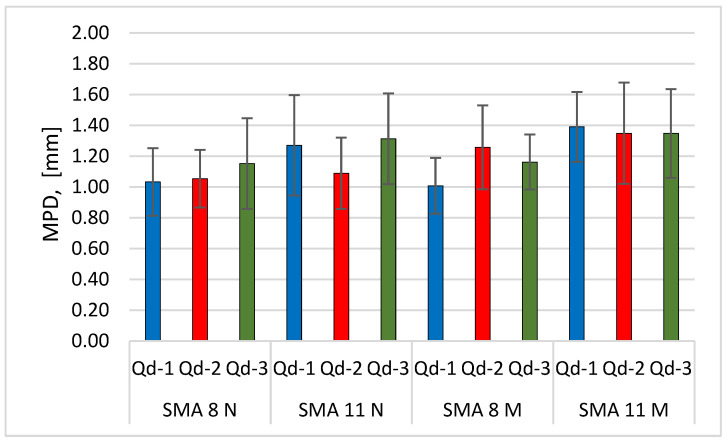
Comparison of the average MPD of the tested surfaces at the end of the glassblasting.

**Figure 29:**
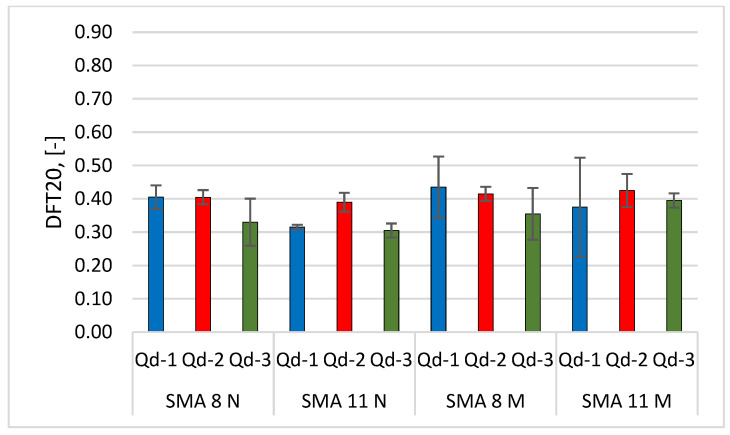
Comparison of the average DFT20 of the tested surfaces at the end of the glassblasting.

Figure 28 and Figure 29 compare the MPD and DFT20 values of the tested surfaces at the end of the glassblasting.

There are no significant differences in mineral composition between SMA 8 with trachybasalt, gabbro and granite aggregates. The same is true for asphalt mixtures with SMA 11. If the glassblasting reflects surface wear such as that occurring under traffic, the MPD values should be comparable for SMA 8 and SMA 11. The same argument applies to the interpretation of DFT20 results. Their values are closely related to the polishing resistance of coarse aggregates. The PSV values of the individual aggregate types are in the same PSV_50_ category, defined according to [46]. This means that the DFT20 should be comparable. The differences between the mean DFT20 values are insignificant for specimens conditioned at −15 °C. However, there are significant differences in the results between individual specimens, which translate into the standard deviations shown in Figure 28. Additionally, the characteristic matte finish on the majority of the aggregate surface proves that this process does not reflect the phenomenon of polishing.

**Figure 30:**
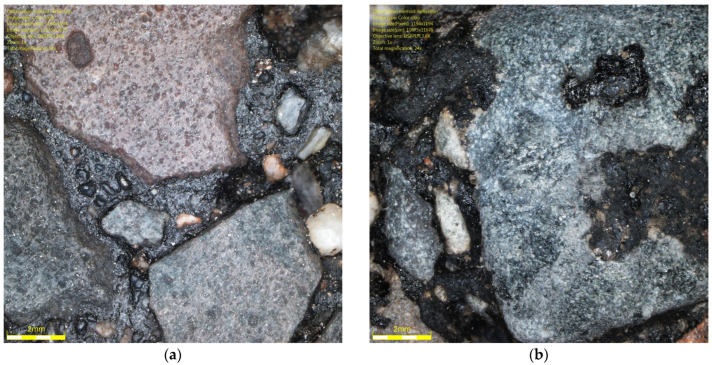
Surface of SMA 11 made with trachybasalt after: (**a**) polishing in FAP; (**b**) glassblasting.

**Figure 31:**
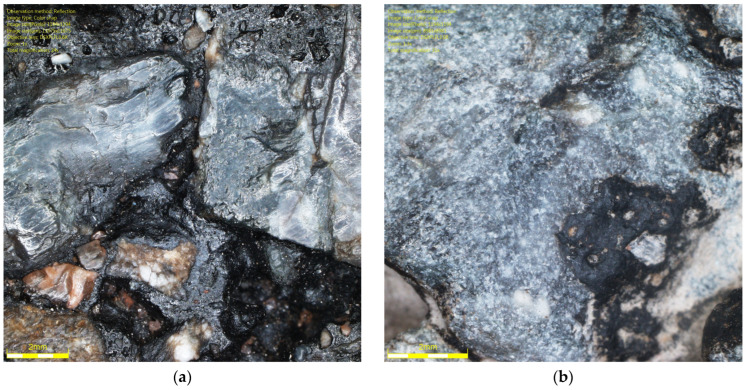
Surface of SMA 11 made with gabbro after: (**a**) polishing in FAP; (**b**) glassblasting.

**Figure 32:**
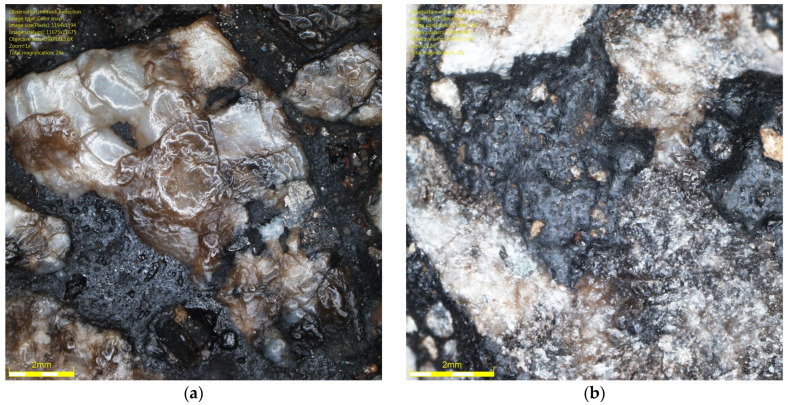
Surface of SMA 11 made with granite after: (**a**) polishing in FAP; (**b**) glassblasting.

To demonstrate the difference between the surface of aggregate subjected to glassblasting and polishing, specimens of the same SMA 11 mixtures were prepared for testing with the FAP device according to EN 12697-49 [47]. This method is well described in the literature [48,49]. A 225 mm diameter specimen of asphalt mixture is subjected to polishing by the action of three rubber cones, water, and quartzite flour. A comparison of images taken under an optical microscope is shown in Figure 30, Figure 31 and Figure 32. The polished surfaces are ring-shaped and 60 mm wide. Unfortunately, these dimensions do not allow for measurements with a retroreflectometer, DFT, and CTM. However, the differences are visible in the images. The effect of the change in microscopic protrusions of rocks due to polishing is areas with a glassy luster. This simulates a phenomenon that occurs in situ, which is responsible for changes in microtexture and causes differences in light reflection across the cross-section of the traffic lane. Friction coefficients are lower in wheel tracks than in the center of the belt. These surfaces should be the basis for luminance assessment. Due to the fact that the dimensions of the specimens intended for testing in the FAP do not allow for comprehensive measurements of the luminance coefficient in scattered light Q_d_, DFT20, and MPD, further research should develop a method that takes into account both aspects related to the simulation of phenomena influencing texture changes and the use of stationary devices used in in situ conditions for measurements, i.e., a retroreflectometer, CTM, and DFT20.

## 4. Conclusions

The research program carried out to verify the method for luminance of SMA wearing courses allowed for the following conclusions to be drawn:

1. The temperature at which specimens are conditioned has a significant impact on the quality of the obtained results. Conditioning at −15 °C significantly improves the process of removing the binder film from the surface of specimens from the mineral mixture compared to those conditioned at 22 °C.
2. The lowest Q_d_ values were found for specimens with the darkest trachybasalt aggregate (SMA 8 M—53.0; SMA 11 M—51.7 mcd/m^2^/lx) and the highest for specimens with the lightest granite aggregate (SMA 8—63.9; SMA 11—59.8 mcd/m^2^/lx). However, the difference between the luminance coefficients determined on the surface of these aggregates was 62 mcd/m^2^/lx.
3. When measuring the luminance coefficient in diffused light Qd after individual glassblasting cycles, it was valuable to visually inspect the surface macroscopically and microscopically using an optical microscope. This allowed us to determine the intensity and predominance of the effects of phenomena occurring in the aggregate grain areas during glassblasting, taking into account the specimen’s conditioning temperature. The characteristic matte surface indicates that glassblasting does not replicate the polishing process that occurs under actual operating conditions, but merely abrasion.
4. Glassblasting removes the binder film from the aggregate surface, but it does not reflect the processes occurring in real traffic conditions (polishing). Consequently, the surface after glassblasting cannot be used to estimate parameters describing skid resistance and macrotexture.
5. Despite the same surface blasting conditions, the petrographic characteristics of the rock from which the coarse aggregate was produced have a significant impact on the removal of the binder film. It was more difficult to remove the binder from aggregate made of a rock with an open-crystalline structure, such as gabbro and granite, than from a cryptocrystalline rock such as trachybasalt.

The method should reflect road traffic conditions, as changes occurring on the surface during use affect luminance, skid resistance, and macrotexture. Consequently, the procedure must enable the comprehensive verification of surface properties. Glassblasting is a cheap and quick procedure, but the variability of MPD and DFT20 results and microscopic surface observations indicate that it does not accurately reflect surface wear occurring in situ. This can lead to incorrect estimation of the luminance potential of materials intended for the wearing course. Further research is needed to improve the laboratory method for estimating luminance, but this should be done using skid resistance assessment equipment.

## Figures and Tables

**Table 1:** Physical properties of coarse aggregates.

Properties	Test Method	Trachybasalt (Qd-1)	Gabbro (Qd-2)	Granite (Qd-3)
Resistance to polishing, PSV [-]	[32]	53	52	52
Resistance to wear, MDE [%]	[33]	8	12	9
Resistance to fragmentation, LA [%]	[34]	12	23	25
Resistance to freezing and thawing, FnaCl [%]	[35]	1	1	1
Density, ρa [Mg/m^3^]	[36]	2.70	2.90	2.66
Water Absorption, WA24 [%]	[36]	0.5	0.5	0.5
Qd [mcd/m^2^ lx]	[30]	53	83	115

**Table 2:** Aggregate particle size distribution.

Passing Fraction [%]						
Sieves [mm]	SMA 8			SMA 11		
	Trachybasalt Qd-1	Gabbro Qd-2	Granit Qd-3	Trachybasalt Qd-1	Gabbro Qd-2	Granit Qd-3
16	100	100	100	100	100	100
11.2	100	100	100	98	96	98
8	95	92	92	57	56	57
5.6	54	55	44	41	41	36
2	25	24	23	25	24	20
0.125	11	9	11	11	10	10
0.063	10.3	9.0	10.2	10.3	9.5	8.9

**Table 3:** Volumetric parameters of SMA mixtures.

Parameters	Test Method	SMA8-Qd-1	SMA8-Qd-2	SMA8-Qd3	SMA11-Qd-1	SMA11-Qd-2	SMA11-Qd-3
Density ρmv [Mg/m^3^]	[38]	2.379	2.561	2.356	2.399	2.573	2.396
Bulk density ρbssd [Mg/m^3^]	[39]	2.306	2.475	2.284	2.332	2.490	2.318
Air voids Vm [%]	[40]	3.1	3.4	3.0	2.8	3.2	3.3
Voids filled with Binder VFB [%]	[40]	26.7	26.3	27.2	29.0	27.6	25.6
Voids in mineral aggregate VMA [%]	[40]	4.2	4.6	4.1	3.9	4.4	4.4

**Table 4:** Surfaces of the tested SMA 8 M and SMA 11 M mixtures after glassblasting.

	8 mm	11 mm
**SMA with trachhybasalt (Q_d_-1)**	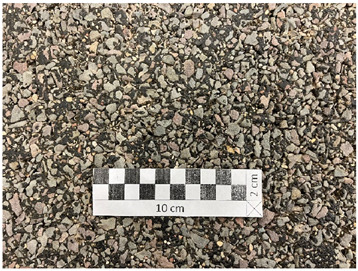	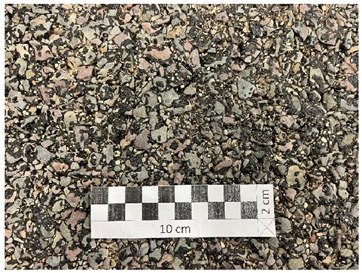
**SMA with gabbro (Q_d_-2)**	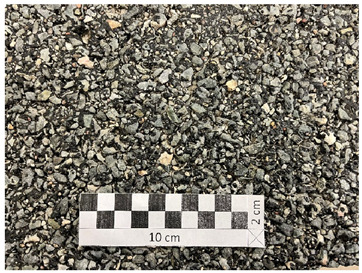	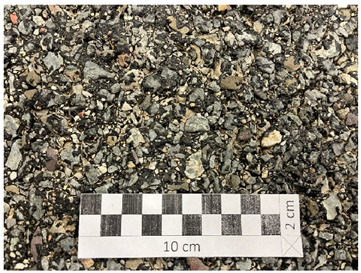
**SMA with granite (Q_d_-3)**	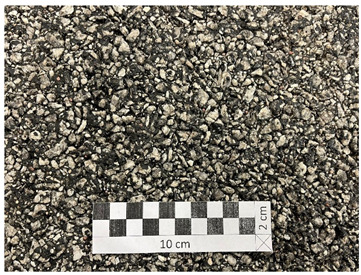	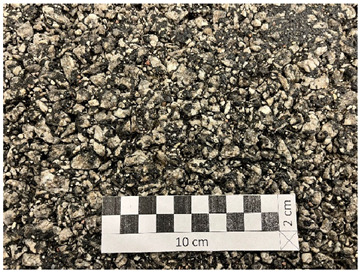

## Data Availability

The original contributions presented in this study are included in the article. Further inquiries can be directed to the corresponding authors.
